# Integrating a Strategic Framework to Improve Health Education in Schools in South Tyrol, Italy

**DOI:** 10.3390/epidemiologia5030027

**Published:** 2024-07-09

**Authors:** Christian J. Wiedermann, Patrick Rina, Verena Barbieri, Giuliano Piccoliori, Adolf Engl

**Affiliations:** 1 Institute of General Practice and Public Health, Claudiana—College of Health Professions, 39100 Bolzano, Italy; 2 Department of Public Health, Medical Decision Making and Health Technology Assessment, University of Health Sciences, Medical Informatics and Technology, 6060 Hall in Tirol, Austria

**Keywords:** mental health education, resilience building, screen time management, gender-sensitive education, health curriculum development, integrated health education, student well-being, post-pandemic, school health programs, South Tyrol, health education

## Abstract

This narrative review addresses the integration of health education into school curricula in South Tyrol, an Italian province with significant cultural and linguistic diversity. This review’s objective is to analyze current health education initiatives and propose a strategic framework to enhance school-based health education, aiming to improve student well-being post-pandemic. The review synthesizes global examples and recent local studies, highlighting the importance of comprehensive teacher training, mindfulness-based interventions, culturally sensitive health education, and community engagement. The key findings indicate that current health education programs in South Tyrol are insufficient to meet immediate public health needs, such as low vaccine uptake and mental health challenges exacerbated by the COVID-19 pandemic. The proposed strategic framework seeks to align educational strategies with the diverse needs of South Tyrol’s student population, thereby improving health literacy and behavior and strengthening the region’s public health infrastructure.

## 1. Introduction

The COVID-19 pandemic has highlighted the critical importance of health education in schools [1,2,3], especially in regions with significant cultural and linguistic diversity such as South Tyrol, Italy [4]. The profound impact of the pandemic on the mental and physical health of youth necessitates a comprehensive approach to health education to promote resilience and well-being [5,6]. Experts have advocated for the introduction of health education as a formal school subject to enhance health literacy and preventive health measures [7]. In South Tyrol, this need is compounded by unique challenges, including vaccine hesitancy influenced by historical mistrust of national authorities, particularly among the German-speaking population [8].

This narrative review aims to analyze the current state of health education in South Tyrol and propose a strategic framework to enhance school-based health education, ultimately improving student well-being post-pandemic. By synthesizing global examples and recent local studies, this review highlights the importance of comprehensive teacher training, mindfulness-based interventions, culturally sensitive health education, and community engagement.

The primary objective of this review is to develop and propose a strategic framework for enhancing school-based health education in South Tyrol. This framework aims to address the specific health education needs of students in the region, considering the unique challenges posed by the COVID-19 pandemic. The guiding research question is as follows: how can current health education initiatives in South Tyrol be enhanced through a strategic framework to improve student well-being post-pandemic?

This study will provide an examination of the current health education initiatives in South Tyrol and develop a strategic framework that aligns educational strategies with the diverse needs of the student population. By addressing these needs, the proposed framework seeks to improve health literacy and behavior, thereby strengthening the region’s public health infrastructure.

## 2. Framework

South Tyrol, or Alto Adige, is a region characterized by cultural and linguistic diversity. It is located in Northern Italy, bordering Austria and Switzerland. This area is unique in its autonomous status, which allows it to maintain its cultural and linguistic roots in Austria, alongside Italian- and Ladin-speaking communities [9]. The region’s population is roughly divided into 70% German-speaking, 25% Italian-speaking, and 5% Ladin-speaking communities. Each of these groups has distinct cultural practices and attitudes towards health [10], which necessitates tailored communication strategies. This diversity poses unique challenges for public health initiatives, particularly programs where communication must be adapted to different cultural norms and languages [4,8].

The multilingual composition of the region not only influences public health communication but also plays a role in addressing broader health challenges, including mental health issues that have persisted among children and adolescents after the COVID-19 pandemic [11]. The diverse cultural and linguistic environment of South Tyrol requires adapted educational strategies that go beyond traditional health topics to include mental health awareness, management of screen time, and promotion of physical activity [12,13]. Vaccine hesitancy remains a critical issue. For instance, a survey conducted in 2021 revealed that only 50% of the German-speaking population was willing to receive the COVID-19 vaccine, compared to 75% of the Italian-speaking population [14].

Health education, as defined by the World Health Organization, includes providing essential knowledge and skills for health promotion and training individuals to actively engage in their health needs and goals [15].

Integrated curriculum development in health education draws on Bronfenbrenner’s Ecological Systems Theory and the Health Belief Model. Bronfenbrenner’s theory emphasizes the influence of multiple environmental systems on child development and highlights the role of schools as critical microsystems that can promote health literacy and behaviors [16]. The Health Belief Model, which explains health behaviors in terms of individuals’ perceptions of risks and benefits, underscores the importance of tailored educational interventions to promote proactive health decisions [17]. Combining these frameworks, integrated curriculum development in health education seeks to create a holistic learning environment that addresses not only academic content but also the socio-emotional and physical well-being of students, thereby promoting a resilient and health-literate youth population.

## 3. Methods

This study uses a narrative review methodology to analyze the current state of health education in South Tyrol and to propose a strategic framework for improving school-based health education. The narrative review approach allows for a comprehensive examination of the existing literature, including both global examples and local studies, to provide a contextual understanding of health education practices and challenges in the region.

Articles were selected based on their relevance to health education, resilience building, and public health in school settings, with priority given to studies conducted in South Tyrol, Northern Italy, or similar multilingual and multicultural regions to ensure contextual relevance. Studies included both qualitative and quantitative research. The review focused on identifying key issues and best practices in health education, such as mental health education, vaccine uptake, screen time management, and gender-sensitive education. 

Global examples of successful health education programs were included in the analysis based on their proven effectiveness and potential applicability to the South Tyrolean context. Local studies and data from South Tyrol were analyzed to identify the unique challenges and opportunities within the region, taking into account the impact of cultural and linguistic diversity on health education initiatives.

A non-systematic search was conducted using PubMed with search terms “(((health education) OR (health literacy)) AND (school)) AND (“South Tyrol” OR Bolzano)” on 19 June 2024. Relevant data were extracted from selected articles, focusing on study objectives, methods, and outcomes. This search yielded 218 hits, of which 5 articles reported on school-based health education topics relevant to the context of South Tyrol, illustrating the scientific activities and contributions in this field within the region.

## 4. Results

### 4.1. Specific Public Health Findings from Recent Studies and Surveys

The COVID-19 pandemic has left a lasting mark on the mental health of youth in South Tyrol, particularly among adolescents. Two prominent challenges that emerged during and after the pandemic—persistent mental health problems and vaccine hesitancy—illustrate the need for change [4,8,11]. While the immediate crisis highlighted low vaccine uptake and the need for acute psychosocial support for youth, it also revealed gaps in health literacy and preventive measures. These challenges can be addressed through school-based health education strategies that not only focus on immediate needs but also build long-term resilience and health literacy among teachers and students. In response to the changing needs of adolescents and young people, other countries worldwide have significantly increased their commitment to school-based health education, recognizing the essential role that such initiatives play in addressing the health challenges facing young people today [18]. This proactive approach aims to enhance the resilience and well-being of youth by equipping them with crucial knowledge and skills to effectively navigate mental health issues and other emerging health risks.

#### 4.1.1. Mental Health Challenges among Adolescents

During the COVID-19 pandemic, South Tyrol, like other parts of the world, experienced an increase in mental health challenges, particularly among adolescents. Barbieri et al. [11] highlighted the persistence of high levels of psychosocial problems among children and adolescents in the region. This study showed that the psychological impact of the pandemic may have long-lasting effects on young people’s mental well-being.

To effectively address these persistent challenges, one possibility is to integrate a comprehensive health education curriculum into schools. This curriculum should include not only traditional health topics but also mental health awareness, coping mechanisms, and resilience-building strategies [13]. As for the above-cited findings, emphasizing the importance of physical activity and the effective management of screen time is also critical, as these factors significantly influence mental health outcomes. The South Tyrol studies also pointed to significant gender differences in the prevalence of mental health problems. Strategies that recognize and address these disparities are essential to ensure that all students, regardless of sex, receive appropriate support and resources to manage their mental health.

Awareness of and response to the mental health challenges faced by students were heightened during the pandemic, with a focus on resources for the provision of psychosocial support and therapy. In addition, a proposal to integrate comprehensive health education into the school curriculum was published and communicated to policymakers during this critical period [6]. However, as the immediate effects of the pandemic begin to subside, there is little visible motivation to implement broader educational reforms. Measures to improve therapeutic services should adequately address the long-term importance of concurrent health education as a fundamental element of mental health support. Maintaining this dual approach is necessary to build lasting resilience and ensure that students are equipped to face current and future challenges.

#### 4.1.2. COVID-19 Vaccine Uptake in Schools

The primary public health problem in South Tyrol is vaccine hesitancy [3]. It is influenced by historical, cultural, and religious factors, dating back to the early 19th century [19], when figures like Andreas Hofer opposed vaccination, fearing it would introduce Protestantism into the Catholic region [20]. During the pandemic, school-based vaccination offers were largely unsuccessful. Challenges included the limited availability of teachers during visits and some teachers’ reluctance to vaccinate. This hesitancy extends beyond COVID-19 to include other vaccines, such as those for human papillomavirus. 

Prevention, as part of the nationally promoted “social education” curriculum, could include comprehensive teaching on vaccination, particularly in South Tyrol. Given that vaccination decisions are primarily parental, it is important to support parents through education rather than place undue responsibility on teachers. By integrating vaccine education into the curriculum and providing culturally sensitive information, schools can help improve vaccine acceptance, ultimately enhancing public health outcomes in the region.

#### 4.1.3. Teachers’ Emotional Distress

Families are the primary environment for children’s well-being, but parents are increasingly overwhelmed by challenges such as single parenthood and work–life balance. This has led to the tendency to delegate more responsibility to schools. However, this shift can place an undue burden on teachers, who are expected to act as experts in areas beyond their primary educational roles, including health promotion and civic education.

Additionally, the increasing number of students from migrant backgrounds poses new challenges. These students, now significantly present in primary schools and expected to reach all levels in the coming years, highlight the need for health literacy to be a key focus for all families. Addressing these challenges requires comprehensive support systems that include both schools and families to effectively promote health education.

Keim et al. [21] described the significant emotional distress experienced by teachers in South Tyrol during the pandemic, including depression, anxiety, and stress, which negatively affected their ability to effectively teach. Nevertheless, studies and anecdotal reports have supported the need for improved health education. Improving health education curricula and promoting collaboration between health professionals and educational institutions could help close the gaps in vaccine literacy and acceptance. Effectively addressing these issues requires comprehensive educational strategies that integrate health education into broader educational initiatives. It is important to provide teachers with the necessary support and resources to effectively manage these additional responsibilities.

### 4.2. Evidence for the Benefits of Improving School-Based Health Education

School-based health education programs are important to address several health challenges, including mental health literacy and vaccination competence. Research highlights the direct benefits of improved health education on students’ mental health as well as the indirect benefits of improved health literacy among teachers and their interactions with parents. However, the variability in program implementation can affect the effectiveness of these initiatives. Therefore, strategic planning and ongoing professional development for teachers are essential, as highlighted by Saboga-Nunes et al. [22], who noted the impact of health literacy on children’s nutritional status and overall well-being, and emphasized the need for consistent and comprehensive educational approaches.

#### 4.2.1. Mental Health through School-Based Education

Improving school-based health education can improve both the direct and indirect determinants of students’ mental health [5]. School-based health education can beneficially affect students’ knowledge and attitudes toward disease prevention, thereby supporting better mental and physical health outcomes [23]. Secondary school students who participated in elective psychology courses demonstrated significantly higher levels of mental health literacy; this increase in literacy not only improved their understanding of mental health issues but also reduced the stigma associated with seeking professional help [24]. A significant benefit of mental health education was also demonstrated for rural children in China, with significant improvements in life satisfaction and self-confidence following a 16-week intervention, in contrast to the decline in similar measures in the control group [25]. These findings highlight the role of structured mental health education in improving the psychological well-being of children and adolescents.

Improving health literacy through school-based programs has a direct impact on students’ ability to manage health information and make informed decisions. Findings from Australia demonstrate the potential benefits of school-based online interventions in delaying harmful behaviors such as alcohol use among adolescents [26]. However, the lack of long-term effectiveness in broader mental health and substance use disorders highlights the need for continuous and adaptive health education strategies in schools to sustain these benefits over time. Evidence from recent systematic reviews, including Porter et al. [27], underscores the effectiveness of mindfulness-based interventions tailored to different school developmental stages. These programs not only directly improve students’ mental health literacy but also indirectly benefit the educational environment through improved teacher engagement and parent–teacher interactions. Porter et al. emphasized the need to adapt health literacy programs to students’ developmental needs, which resonates with the findings of significant improvements in social and emotional skills, attitudes, behaviors, and academic performance following structured social and emotional learning programs [28]. This finding suggests that structured, age-appropriate health education is critical for maximizing the effectiveness and consistency of implementation.

Kealy-Ashby et al. [29] found that pre-service teachers who received health literacy training felt more competent and confident in their teaching abilities, which could translate into better student support and better educational outcomes. In addition, the indirect effects of school-based programs, such as improved teacher health literacy and increased parent–teacher collaboration, contribute to a supportive educational atmosphere, which is essential for students’ overall well-being [27,28]. 

#### 4.2.2. Addressing Vaccine Hesitancy

Teachers can integrate vaccine education into their curriculum and counter misconceptions using factual science-based information tailored to different cultural groups. Teachers also build strong relationships with their families, enabling them to foster dialogue regarding immunization, address concerns, and provide credible resources to build community confidence in vaccines. The critical importance of teacher training and family involvement in health education, particularly around immunization, is underscored by the results of the VACUNASEDUCA study [30]. This study involved 1000 participants from 76 countries across a range of sectors and used a descriptive cross-sectional design with a quantitative approach. The results showed that effective health education and the proper use of vaccines were significantly influenced by well-trained teachers and informed families.

### 4.3. Current State of School-Based Health Education and Research

The pandemic has highlighted significant gaps in the health education system, particularly in addressing mental health issues and vaccine hesitancy. Enhanced education programs are needed to support students’ recovery and build resilience to future public health crises. A 2016 systematic review of the literature revealed a significant gap in health education research in Italian schools [31]. With only five randomized control trials conducted between 1983 and 2016 focusing on school and family settings for cardiovascular health promotion, the study highlights a significant lack of validated interventions to address risk factors from an early age in Italy. This lack of focused research and program development underscores the need for increased effort to implement effective science-based health education programs that can significantly improve the health outcomes of Italian children.

The Early Vascular Aging–Tyrol study examined cardiovascular health (CVH) behaviors among adolescents in different educational settings in Tyrol, Austria, and South Tyrol, Italy [32]. Significant disparities were found based on school type. Apprentices and vocational students had less favorable CVH behaviors, with apprentices showing the highest prevalence of non-ideal BMI (16.2%) and smoking habits (47.9%). High school students had lower rates of both unfavorable BMI (4.2%) and smoking. The study emphasized the need for targeted interventions to address these disparities.

The “Smuovi la Salute” study focused on health education for children from migrant and low-socioeconomic-status backgrounds in Northern Italy, including South Tyrol [33]. Significant disparities in health behaviors were found among different school types. Primary school children in areas with high migrant and low-socioeconomic-status populations showed notable improvements in nutritional knowledge and physical activity. However, the intervention’s impact varied, with greater knowledge gains but more significant behavior change challenges in schools with higher proportions of low-socioeconomic-status or migrant backgrounds.

The ‘Preparation, Education. Action, Coping, Evaluation’ (P.E.A.C.E.) pack intervention in Northern Italy demonstrated significant effectiveness in reducing victimization and improving peer support among severely bullied high school students [34]. The P.E.A.C.E. pack program’s success was corroborated by Lazuras et al. [35], emphasizing its positive effects on self-efficacy and peer relationships among students. Brighi et al. [36] highlighted the critical role of resilience and direct confrontation in mitigating the emotional impacts of cybervictimization, showing that these strategies enhance adolescent well-being. Together, these studies underscore the importance of targeted, resilience-building interventions in addressing both traditional bullying and cyberbullying in school settings, and collectively demonstrate the active efforts and scientific contributions towards improving school-based health education in South Tyrol.

#### 4.3.1. Overview of Health Education Initiatives in South Tyrol

According to a 2010 document on health education in schools of the German-speaking population in the Autonomous Province of Bolzano, the development of life skills and the prevention of addiction, violence, and suicide are emphasized from kindergarten to high school [37]. This advocates a holistic approach to development that integrates pedagogical, psychological, educational, and sociological perspectives. This document encourages interdisciplinary collaboration among school leaders, teachers, and external consultants to effectively tailor health education to local needs. The specific initiatives highlighted include the creation of supportive school environments and the use of established programs, with an emphasis on the personalization of health education. The challenges identified in 2010 included varying levels of acceptance among teachers and the need for consistent content across schools [37], with calls for strategic planning and comprehensive support systems.

##### ‘Health-Promoting School’ Program

In 2010, South Tyrol adopted the ‘Health-Promoting School’ program from Austria [38]. This initiative aimed to certify schools as health-promoting, based on specific criteria. Schools were required to integrate health promotion projects into their three-year plans, implement these projects, and evaluate their outcomes. Successful schools were awarded a ‘Health-Promoting School’ certificate. The program ran for approximately ten years, during which many schools received the certificate, partly incentivized by funding opportunities [39]. Schools used the funds to create dedicated areas in staff rooms for relaxation and purchase exercise equipment for daily use. Different schools have focused on various aspects of health promotion such as exercise and mental well-being [40].

In a new document describing the quality evaluation framework of public schools for the 2022–2023 school year in the Autonomous Province of Bolzano, health education was not mentioned as a component of the evaluation criteria [41]. The absence of health education in quality assessments underlines the need to better integrate health literacy into the curriculum to ensure comprehensive educational assessments that are aligned with broader public health goals.

##### Project-Based Health Promotion

Health promotion in South Tyrolean schools is largely project-based, with schools having the autonomy to choose their own health projects. These projects cover a wide range of topics, from healthy eating and physical activity to mental health and environmental protection [40]. Health education lessons frequently are not programmed in advance; instead, they are often conducted during canceled hours when teachers are absent or unavailable, involving activities such as cooking, excursions, and sports events. Despite the diverse range of topics, the lack of a standardized curriculum leads to uneven health-related knowledge among students.

In this context, several key points were highlighted in the interviews with high school teachers responsible for health promotion, as summarized in Table 1. These points underscore the necessity for systematic integration and enhancements of health promotion in daily school life to support not only the physical but also the mental health of students.

#### 4.3.2. Teacher Involvement and Training in Health Education

##### University Training

Academic training for kindergarten and primary school teachers is managed locally by a region-specific academic institution. The Free University of Bolzano/Bozen offers a course titled ‘Management of Emergency Situations with Children, Acute and Chronic Illnesses in Kindergarten and School’ as an optional subject of the ‘Master’s Degree in Educational Science for the Primary Level’ [42]. A 20 h course is available for all three official languages in the region. The curriculum covers a wide range of topics, including first aid at all ages, physiological and pathological changes in children during illness, legal frameworks in educational settings, and preventive health measures, such as immunization. The 20 h course is optional and the only health education course offered in the program.

The scenario changed significantly for middle and high school teachers. These levels of education require a university degree, which cannot be obtained under the direct supervision of South Tyrol. Consequently, prospective teachers at these levels often attend universities in other parts of Italy or in German-speaking countries abroad. This decentralization means that the responsibility for health education training lies largely in the universities where teachers are trained, outside the direct control of South Tyrol. In Austria, there are university teacher-training courses for the most important school subjects, which are sufficient for teaching in South Tyrol. Diploma studies without a teaching degree, such as those completed in Italy, require supplementary in-service teacher training amounting to 30 European Credit Transfer and Accumulation System points (ECTS).

The 30 ECTS can be completed either through university courses or organized by local educational authorities with university lecturers. This includes practical didactic training. Their current approach integrates university coursework with practical training conducted by field-experienced teachers and was found to be highly effective. The health education topics were not sufficiently covered.

##### Continuing Education

Teachers’ involvement in health education has been inconsistent. For middle and high school teachers, continuing health education is available, but it has not been systematically integrated into their initial training. Instead, it is offered on a project-by-project basis, linked to ongoing health education projects in schools. Participation in these training programs was voluntary rather than mandatory. This arrangement poses challenges in ensuring that all teachers have consistent and comprehensive training in health education, which could affect the effectiveness of health education initiatives in schools in South Tyrol. As a result, professional development in health education remains an adjunct to formal teacher-training programs rather than a fundamental component.

Continuing education for teachers in South Tyrol is not part of the coordinated curriculum. Currently, the training follows a state plan. Key programs include “Weatherproof” for middle and upper schools, “Becoming Strong Together” for elementary schools, and the “We Project”, which focuses on social learning, self-confidence, mindfulness, and social interaction. These programs are designed to be conducted by experts who visit schools and collaborate with teachers. However, in practice, some criticize that experts may limit their involvement to email communications. Despite these challenges, a “multiplier system” has been developed, enabling trained teachers to independently offer these programs in their schools, which has been generally well received by those able to participate effectively.

#### 4.3.3. School Social Pedagogues Support System

To address the increasing psychosocial issues among students, South Tyrol introduced the role of social pedagogues in German and Ladin schools. These professionals work on site and collaborate with external psychologists who visit schools periodically. This model ensures that social pedagogues are integrated into the school environment, thereby providing continuous support. In contrast, the Italian Directorate of Education in South Tyrol does not have this specific role and relies more on external psychologists claiming this approach provides a clearly defined expert as a contact point. In surveys on mental health [11], some parents of students in German schools have expressed the need for more direct access to external experts. Despite the permanent presence of social pedagogues, these professionals may be seen as lacking the neutrality of an external consultant. The implementation of school social pedagogues was initially funded through the European Social Fund, and due to positive experiences, this role has been institutionalized in elementary, secondary modern, and middle schools. Almost all school directorates currently have at least one social pedagogue. Although they do not directly teach academic subjects, they are trained to provide guidance in the management of psychosocial projects, assist with everyday challenges, and promote social integration and the personal growth of students, particularly those in challenging circumstances or with special needs.

### 4.4. Strategic Framework for Enhancing Health Education in South Tyrol’s Schools

The current system faces several challenges, including reluctance to introduce health education as a separate subject due to concerns about curriculum overload. Teachers are often required to juggle multiple responsibilities, which can dilute their effectiveness in providing support and building relationships with their students. The interdisciplinary approach to social education has been a positive step, but the overall situation remains heterogeneous, with schools having varying levels of engagement and success in health promotion.

To improve health education, it is essential to standardize training and ensure that health promotion is consistently embedded across all schools. Increasing the number of social pedagogues in schools as well as external psychologists and ensuring sustainable funding for their positions are critical steps. Additionally, measuring health literacy among teachers and students can help identify areas for further training and support.

#### Social Education as Interdisciplinary Learning Area

Recently, rather than introducing a new subject, South Tyrol incorporated health promotion into an interdisciplinary learning area known as ‘social education’ [43]. Initially referred to as ‘civic education,’ the social education curriculum in South Tyrol is designed to promote holistic development by integrating various educational themes, such as personality and social skills, cultural awareness, law and politics, economy and finance, sustainability, health, mobility, and digitalization. This interdisciplinary approach promotes competency-based learning and project-based activities, allowing students to engage in meaningful real-world applications of their knowledge. Schools develop specific curricula within these guidelines to promote holistic education that prepares students for active and informed citizenship. They are free to individually select topics and coordinate transversally among disciplines, ensuring that the chosen themes are integrated seamlessly across various subjects. This flexibility allows schools to tailor their health education efforts to meet the unique needs of their student populations while fostering interdisciplinary learning and collaboration [43].

Health education within the framework of social education emphasizes the development of comprehensive health literacy among students. It includes mental, physical, preventive, media literacy, and sexual health [43]. By integrating health topics across subject areas and using project-based learning, the curriculum aims to promote healthy lifestyle choices, mental and emotional well-being, and critical thinking regarding health information. Collaboration with health professionals ensures that students receive accurate and relevant knowledge and are empowered to make informed decisions about their health.

This approach integrates health education into a broader curriculum, without overloading students with additional subjects. However, for this transversal health education to be effective, it is crucial to align health education teaching with a larger number of teachers. Consistent and coordinated efforts across different subjects and teachers are necessary to ensure that students receive a comprehensive and cohesive health education.

### 4.5. Proposed Educational Initiatives for South Tyrol

**Figure 1:**
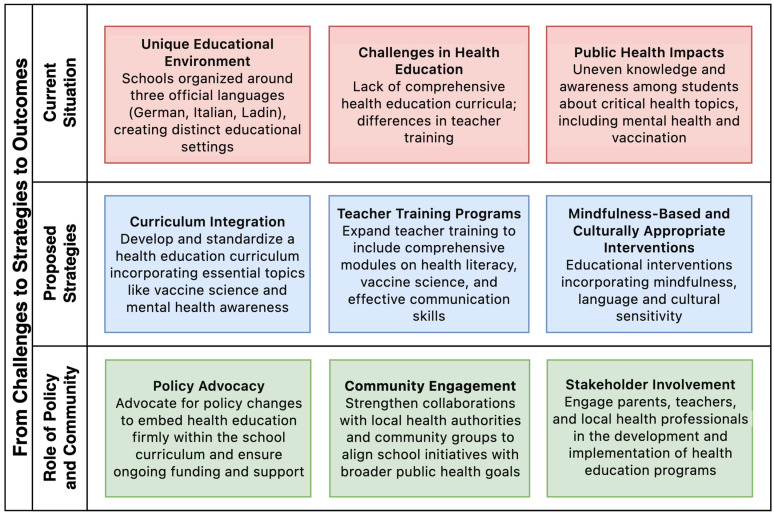
Strategic framework for enhancing health education in South Tyrol’s schools.

In South Tyrol, schools operate in the German, Italian, and Ladin languages, creating a unique educational environment [44,45]. Tailoring health education to these diverse linguistic communities presents challenges, particularly in terms of teacher training and curriculum development. Addressing these issues requires comprehensive strategies to improve teacher training, develop inclusive health education curricula, and utilize multilingual education programs to overcome language barriers. These efforts are essential for improving public health outcomes and preventing vaccine hesitancy (Figure 1).

#### 4.5.1. Integrating Health Issues into the Curriculum

Given the ongoing need for improvement [37], particularly in the areas of prevention and mental health (including media and screen time literacy), it is imperative that these topics are integrated into the curriculum. A structured, mandatory school-based health education curriculum should be developed based on the Health Promoting School Program. This curriculum must include comprehensive modules on vaccine science, disease prevention, mental health awareness, and media literacy tailored to the specific cultural and linguistic context of South Tyrol.

#### 4.5.2. Project-Based and Transversal Teaching Approach

Building on the development of project-based health education, a transversal approach should be adopted in social education. This approach integrates health issues across subjects and ensures that health education is embedded in everyday school life. The cross-curricular learning area of social education includes specific health education modules that comprehensively address prevention and mental health.

#### 4.5.3. Teacher-Training Programs

Teacher-training programs should be strengthened to support this integrated approach to health education. Current health education training, which is limited to elective lectures [42], should be expanded into mandatory, comprehensive modules. These modules should cover health literacy, vaccine science, mental health, and effective communication skills to enable teachers to deliver health education confidently to diverse student populations.

#### 4.5.4. Culturally and Linguistically Appropriate Mindfulness-Based Interventions

Educational interventions should be culturally and linguistically appropriate considering the diverse beliefs and values of South Tyrol. The materials must be designed to be accessible and appealing to all students. Adapting frameworks such as the German Health Literate School Project [46] can provide robust standards for integrating health literacy into school development plans, ensuring that health literacy becomes a core part of the educational process.

#### 4.5.5. Policy and Community Engagement

Policy advocacy is essential to embed health education in the school curriculum [5]. This includes securing funding, revising educational standards to include health education as a core subject, and ensuring a rigorous assessment. Collaboration between schools, health authorities, and the community is crucial for the success of health education programs [12]. Regular community meetings, parental involvement, and partnerships with local health clinics can increase program relevance and effectiveness. 

By integrating these strategies—comprehensive curriculum development, enhanced teacher training, culturally sensitive materials, supportive policies, and active community engagement—South Tyrol can improve health literacy, increase immunization rates, and achieve better public health outcomes.

## 5. Conclusions

South Tyrol is at a critical juncture in its approach to public health and education. The unique cultural and linguistic diversity of the region, while being a source of richness, also presents specific challenges that require tailored solutions, particularly in the context of health education. The COVID-19 pandemic has highlighted the urgent need for comprehensive school-based health education that covers a wide range of health issues, including mental health and vaccine literacy.

The proposed strategic framework for health education in South Tyrol consists of several key elements: comprehensive teacher training, mindfulness-based interventions, culturally sensitive health education, and active community engagement. These components are designed to create a holistic learning environment that addresses both the academic and socio-emotional needs of students.

Evidence from global and local studies underscores the effectiveness of well-structured health education programs in improving mental health literacy, reducing stigma, and enhancing student well-being. For instance, studies conducted in rural China and secondary schools in Slovakia have demonstrated significant improvements in mental health outcomes and health literacy among students, providing a strong foundation for similar initiatives in South Tyrol.

To ensure long-term resilience and health, South Tyrol should integrate these elements into the school curriculum, supported by ongoing teacher training and community engagement. This integration aims to address immediate health challenges and promote a lasting culture of health literacy and prevention. Support from policymakers, educational leaders, and the community is critical for transforming these initiatives into sustainable actions that benefit all students, regardless of their linguistic or cultural background.

In conclusion, by committing to this strategic framework, South Tyrol can strengthen its public health infrastructure, better prepare its youth to face health challenges, and build a more resilient future for all its residents. The lessons learned from the pandemic provide a blueprint for transforming health education to meet the needs of diverse student populations in multilingual and multicultural regions.

## Figures and Tables

**Table 1:** Overview of health education initiatives and challenges in South Tyrolean schools in 2024.

Key Point	Description
Designation and Commitment	Public schools have designated teachers responsible for health promotion projects. These teachers also participate in elective training sessions that focus on health promotion and education.
Participation in Training	Training events on health education are poorly attended, typically only by the teachers designated as responsible for health promotion. There is a lack of broader engagement among the entire teaching staff.
Project Selection and Diversity	Schools have the freedom to choose their health projects, leading to a wide range of topics. These range from classic health promotion topics such as healthy eating and physical activity to environmental protection and climate change. First aid classes are also available. External experts are brought in for specialized topics like internet safety and mental health.
Implementation in School Routine	Health education lessons frequently are not programmed in advance. They can include up to 20 h per year per class, involving activities such as cooking, excursions, and sports events.
Disinterest Among the Majority of Teachers	A significant number of teachers show little interest in actively participating in health education, sticking to their subject areas and not attending relevant training sessions.
Response to Psychosocial Problems	Due to the increasing psychosocial problems among students, there has been a reorganization of support systems in schools. Every school now has access to social pedagogues, school psychologists, and improved structures for psychosocial support.

## Data Availability

No new data were created or analyzed in this study. Data sharing was not applicable to this study.
